# Patient and Provider Attitudes and Preferences Regarding Early Palliative Care Delivery for Patients with Advanced Gastrointestinal Cancers: A Prospective Survey

**DOI:** 10.3390/curroncol31060253

**Published:** 2024-06-13

**Authors:** Oren Levine, Daryl Bainbridge, Gregory R. Pond, Marissa Slaven, Sukhbinder Dhesy-Thind, Jonathan Sussman, Ralph M. Meyer

**Affiliations:** 1 Department of Oncology, McMaster University, Hamilton, ON L8S 4L8, Canada; bainbridgd@hhsc.ca (D.B.);; 2 Department of Family Medicine, McMaster University, Hamilton, ON L8S 4L8, Canada

**Keywords:** palliative care, models of care, primary care, gastrointestinal cancer, integrated care

## Abstract

Early integrated palliative care (EIPC) for patients with advanced cancers requires the involvement of family doctors (FDs) and oncologists. We compared attitudes between patients and their providers regarding the delivery of EIPC. Patients with newly diagnosed incurable gastrointestinal (GI) cancer at a tertiary cancer centre in Ontario, Canada, were surveyed using a study-specific instrument regarding the importance of and preferences for accessing support across eight domains of palliative care. Physicians within the circle of care completed a parallel survey for each patient. The concordance between patient and physician responses was analyzed. A total of 66 patients were surveyed (median age 69, 35% female). All had an oncologist, 12% had a specialist palliative care provider (SPC), and 97% had an FD, but only 41% listed the FD as part of the care team. In total, 95 providers responded (oncologist = 68, FD = 21, SPC = 6; response rate 92%; 1–3 physician responses per patient). Disease management and physical concerns were most important to patients. Patients preferred to access care in these domains from oncologists or SPCs. For all other domains, most patients attributed primary responsibility to self or family rather than any healthcare provider. Thus, concordance was poor between patient and physician responses. Across most domains of palliative care, we found low agreement between cancer patients and their physicians regarding responsibilities for care, with FDs appearing to have limited involvement at this stage.

## 1. Introduction

Palliative care providers play an important role in supporting patients with advanced cancer during the course of illness. The World Health Organization (WHO) defines palliative care as “an approach that improves the quality of life of patients and their families facing… life-threatening illness, through the prevention and relief of suffering by means of early identification and impeccable assessment and treatment of pain and other problems, physical, psychosocial and spiritual [1].” This definition is not specific to one care provider, nor is it specific to end-of-life (EOL) care. Although traditionally, palliative care was associated with EOL and was introduced after the discontinuation of disease-modifying cancer therapy, there has been a paradigm shift with a new interest in early integration of palliative care (EIPC) concurrent with standard disease-modifying oncology care. Multiple randomized trials show the benefits of this approach, including improved mood, symptom burden, quality of life, and prolonged survival among patients with various types of advanced cancers [2,3,4,5,6]; however, many of the interventions studied are complex and may not be readily implemented.

Tiered palliative care with family doctors (FD), oncologists and specialized palliative care (SPC) teams engaged according to the complexity of symptom burden has been proposed to meet the needs of all patients with advanced cancer [7,8,9,10,11]. In this model, all clinicians can adopt a palliative approach to care regardless of provider type [12]. Accordingly, SPC is reserved for patients with the most complex symptom burden. However, multiple barriers to realizing the integration of a palliative approach to care in oncology have been identified, including a lack of role clarity between providers, professional reluctance to begin these discussions early in the disease trajectory, and differences in understanding of EIPC between patients and their providers [13,14,15,16].

There is limited empirical literature regarding EIPC involving family physicians, oncologists and SPC practitioners. It is unknown whether different healthcare practitioners agree on respective responsibilities for providing early palliative support to patients with advanced cancer. Facilitating collaborative EIPC requires an assessment of current beliefs among patients and providers. The objective of this study is to explore attitudes regarding optimal delivery of EIPC among patients with advanced cancer, oncologists, family physicians and SPC practitioners. Using a survey involving patients and their providers, we aimed to explore the following questions: (1) In patients with a new diagnosis of advanced gastrointestinal (GI) cancer, what is the perceived importance of different domains of palliative care, and how do patients wish to access support for each? (2) Do patients and their healthcare providers agree on the importance of each care domain, and who the most responsible provider(s) should be for each?

## 2. Materials and Methods

We conducted a prospective survey at the Juravinski Hospital and Cancer Centre (JHCC) in Hamilton, ON, Canada. This is a site within Hamilton Health Sciences, a research-intensive multi-site healthcare institution. The JHCC is a large regional cancer center in southern Ontario, serving approximately 7500 new patients annually. There are approximately 800 new referrals to the GI disease site team (DST) at the JHCC per year, and around 50% have a diagnosis of advanced cancer. The study received ethics approval from the Hamilton Integrated Research Ethics Board (project #3503).

### 2.1. Population

Survey data were collected from patients with advanced gastrointestinal (GI) cancer, their JHCC-based physicians (oncologists and SPC physicians), and their family doctor, responding in reference to the patient’s current care. The patient eligibility criteria were: 18 years of age or older; patient seen in outpatient clinic; confirmed cancer diagnosis of GI origin; advanced stage of malignancy (non-curable based on local extent of disease or distant metastasis that is not amenable to definitive therapy, or patients previously treated for localized cancer who experienced non-curable relapse); able to provide informed consent; and able to answer survey in English (with help from translator if needed). The target time frame for enrolment was within 8 weeks of the first oncology consultation. Patients who consented to study enrolment were asked to list all providers participating in care and for permission to contact/survey these providers. This was carried out to identify clinician recipients of the provider survey, and providers answered survey questions in relation to the specific patient’s care. In this way, physician/patient dyads were created. If more than 1 physician responded to the survey in relation to the same patient, physician/physician dyads were created.

### 2.2. Survey Development

The research team developed unique patient and provider surveys based on the relevant literature and expert opinion from multidisciplinary perspectives. Input on survey design was sought from the Patient and Family Advisory Council associated with the JHCC. Items address the eight domains of illness and bereavement as outlined by the Canadian Hospice Palliative Care Association (CHPCA) [17]. These domains are disease management, physical, psychological, social, spiritual, practical, end-of-life care and death management, and loss and grief. For the purposes of our study on EIPC, we framed the End-of-Life Care domain as Making Plans in Case Your Health Worsens.

The questions on the patient survey asked about patient demographics, and for each of the eight domains: (1) How important is this part of your care currently? (5-point scale, “not at all” to “among the most important”); (2) Who is helping you most with this part of your care? (Medical Oncologist, Radiation Oncologist, Family doctor, Palliative care specialist, Other [specify]); and (3) Who would you like to help you the most with this part of your care? (Same response options).

The provider survey asked questions about provider demographics (e.g., medical role, years of experience, and palliative care training) and questions specifically about the relevant patient, including life expectancy and for each of the eight domains: (1) Importance in palliative care in general (5-point scale, “not at all” to “among the most important”); (2) Relevance to specified patient’s care (same response options); and (3) Provider who should be most responsible for this domain in the patient’s care (each provider type rated on a 6-point scale, “not applicable”, “not responsible” to “most responsible”).

The final surveys were pilot-tested with clinicians from McMaster University representing multiple disciplines, content experts, and patient communication experts to ensure face validity and clarity. Modifications were made according to feedback. The first 10 patients recruited served as a pilot group to test the usability of the surveys and the willingness of all stakeholders to provide timely responses. No issues were identified and therefore these 10 patients’ data were included in the study results.

### 2.3. Recruitment and Data Collection

All new referrals to the GI disease site team (DST) at the JHCC for either medical or radiation oncology consultation were screened for eligibility by the study PI (OL). Final study eligibility was determined by the patient’s oncologist. Patients deemed eligible were approached for study involvement by their responsible oncologist and/or oncology nurse, provided with a study information sheet, and asked for permission to be contacted by the study coordinator. To facilitate this patient approach, (1) the responsible oncologist and nurse were informed (emailed) of the patient’s potential eligibility at the next and subsequent visits (if missed), and (2) a study reminder was affixed to the patient’s chart immediately prior to visit. Study materials and reminders were also provided in clinics for oncologists and nurses to screen/approach potentially eligible patients who had not been pre-screened (e.g., patients experiencing a non-curable cancer recurrence while on surveillance by an oncologist for previous early-stage GI cancer). Ongoing general reminders to consider patients for the study were provided to oncologists by email and at DST meetings.

Eligible patients were further briefed on the study by the coordinator (DB) via phone or in person, and if the patient was agreeable, informed consent to participate was obtained. The coordinator then conducted a structured interview using the patient survey or arranged and completed this at a later time. Standardized explanations of the care domains were provided in the study materials given in advance and described to the participant at the beginning of the survey interview. Patients completing an interview were asked permission to contact the main providers they identified within their circle of care. If permission was granted, these providers were contacted to self-complete the provider survey in reference to the patient. The Dillman Total Design Survey Method was followed to help ensure provider survey completion, with up to six total subsequent email, fax, and phone contacts [18].

### 2.4. Outcomes and Analysis

The primary outcome was defined as the concordance of attitudes among patients and providers regarding provider responsibility towards caring for patients across palliative care domains. For each patient, a specific provider is deemed to have a concordant attitude if they consider the same provider as being most responsible for care within a given domain as the patient. Since some patients had multiple providers identified and responded to surveys, each patient/provider dyad was deemed a unique response.

Secondary outcomes include the concordance of perceived relevance for each palliative care domain between patient and providers, and the concordance in perceived responsibilities between different providers for each palliative care domain.

Descriptive statistics were used to summarize patient and provider characteristics, survey responses and outcomes. Dichotomization of “perceived importance/relevance” and level of responsibility” outcomes was performed to improve statistical interpretation of results. Then, 95% confidence intervals were constructed for outcomes of interest using the Clopper–Pearson method.

### 2.5. Sample Size and Feasibility

The sample size was calculated to ensure that estimates were sufficiently precise to determine the true level of concordance with reasonable accuracy. Hence, it was desired that the half-width of any 95% confidence interval for the true proportion (concordance) was no greater than 0.1. Since maximum variability occurs when the proportion is equal to 0.5, a two-sided, 95% confidence interval has half-width <0.10 with 104 data points. To account for a potential non-response rate of 20%, a sample size of 130 patients was targeted.

## 3. Results

Between October 2017 and November 2018, 111 patients were deemed eligible by their oncologist and approached for the study. However, one of these patients was later determined not to have advanced GI cancer and so was excluded (see Supplemental Figure S1 for the recruitment flow diagram). Of the 110 patients, 25 (22.7%) declined to be contacted about the study. Among the 85 agreeing to be contacted, 15 (17.6%) died prior to completing the survey, and 4 (4.7%) declined to participate when contacted. The patient characteristics for the remaining 66 (60.0%) who completed a survey are summarized in Table 1. Recruitment was slower than expected and we were resourced only for one year of data collection, hence stopped short of the target sample size. The structured interviews with patients took a mean of 30 min. The median time from the first oncology pre-screening assessment for advanced cancer to patient survey completion was 35 days. In some cases, there were longer spans of time between screening and survey completion due to the oncologist waiting until subsequent visits to deem the patient eligible for approach, ongoing testing required to confirm the diagnosis, the patient immediately starting cancer treatment or undergoing surgery following the first oncology consultation, and/or difficulty in contacting eligible patients regarding the study.

Among patient respondents, 23 (35%) were female, the median age was 69 years, and the most common diagnoses were cancer of the esophagus (23%), colon (18%), or pancreas (15%), see Table 1. Advanced colorectal cancer is likely under-represented in the study population because we only screened new referrals for eligibility, and often advanced colorectal cancer is a recurrence of early-stage disease for patients who are already on surveillance at the cancer centre.

The number of healthcare providers per patient ranged from 1 to 3 unique providers. All patients had an oncologist, 8 (12%) had an SPC, and 64 (97%) had a family doctor, but only 27 (41%) identified their family doctor as a participant in current care. Responses (and rates) by provider type were: 68 (100%) oncologist; 21 (78%) family doctor; 6 (75%) SPC. The demographics of healthcare provider survey respondents are summarized in Table 1. Although 17 patients identified a radiation oncologist as part of their healthcare team, in 14 of these cases, the patient had not yet seen this oncologist, for example, brachytherapy scheduled for a future date. As such, only three of these providers were surveyed, being those that had consulted with/treated the patient at the time of data collection.

### 3.1. Perceived Importance of Palliative Care Domains among Patients and Providers

Patients and providers were asked to rate the relevance of each domain of palliative care in reference to the patient’s current status (see Supplemental Table S1 for full patient data and Table S2 for providers’ responses on general importance). Patients most often rated disease management (80.6%, 95% Confidence Interval [CI] = 68.7% to 89.1%) and physical concerns (67.4%, 95% CI = 55.6% to 79.1%) as important domains of their care (i.e., “very important”, or “among the most important parts of care”). Practical (37.9%, 95% CI = 14.5% to 36.4%) and social (36.4%, 95% CI = 25.0% to 49.1%) concerns were identified as important to a lesser extent, whereas psychological (27.5%, 95% CI = 17.0% to 39.6%), spiritual (29.7%, 95% CI = 18.3% to 41.3%), and loss/grief (27.8%, 95% CI = 14.5% to 36.4%) were identified as important domains least often by patients. In comparison, providers’ rating of the relevance of each domain of care (Very relevant or Among the most important parts of care) were as follows: disease management (85.0%, 76.5% to 91.7%) and physical concerns (83.2%, 95% CI = 74.1% to 90.1%), psychological (73.6%, 95% CI = 63.7% to 82.2%), spiritual (25.3%, 95% CI = 16.9% to 35.2%), and loss/grief (31.1%, 95% CI = 22.4% to 41.9%).

The concordance of responses between patients and providers on domain importance (relevance) to the patient’s care is shown in Table 2. Patients and providers often agreed on the importance of disease management (72.0% agreement) and physical concerns (68.6% agreement), with most rating these to be high priorities. Loss and grief had a similarly high level of agreement (70.0%), but most physicians and patients deemed this domain to be somewhat important, minimally important or not at all important. While less than 30% of patients deemed psychological concerns as important, almost three-quarters of physicians deemed this domain to be important. Physicians were more likely than patients to deem social, practical and making plans as important to the patient’s care.

### 3.2. Patient and Provider Perception of Responsibility for Care Delivery across Domains of Palliative Care

Across the domains of palliative care, patients were asked which provider is currently providing care and which provider would be preferred for care. Responses were compared for agreement (See Table 3). There was high patient concordance (>70%) between actual and preferred providers in all care domains, suggesting that patients are largely accessing care according to their preferences. However, some discrepancies were noted in the disease management, physical concerns and psychological concerns domains. Most patients (68.2%) wanted their medical oncologist to be primarily responsible for their disease management, but among these patients, only 84% indicated that their medical oncologist was most responsible, while 15 indicated another person (including a surgeon, family member, self, nurses, and home care).

Many patients also wanted their oncologists to be primarily responsible for their physical concerns (42.4%), while overall indicating a preference for oncologist care <5% of the time in the other care domains. Patients were less likely to desire care from other physician types (FD or SPC) across most of these domains of care (disease management = 7.6%, physical concerns = 18.2%, other <10%); however, >50% of patients indicated they wanted mostly family or no one/self to help them the most with psychological, social, spiritual, practical, worsening health, or loss and grief concerns. These were not itemized response options; rather, patients selected ‘other’ and specified non-clinician options through comments.

Patient/provider dyads were assessed for agreement regarding perceived responsibility for care across palliative care domains. A total of 95 dyads were included (68 patient/oncologist, 21 patient/family doctor, 6 patient/SPC). Patient responses to the question ‘Who would you like to help you the most with this part of your care?’ were compared to provider responses within dyads. Provider responses were considered concordant if the patient-identified preferred provider was also listed as ‘should be most responsible’ by the provider respondent. We found high agreement only in the domain of disease management (67.4%), and physical concerns (47.4%), but agreement was low across all other domains, ranging from 0% to 15.8%, where providers attributed responsibility to a clinician and patients attributed responsibility to self or family member.

### 3.3. Perceptions and Agreement among Providers Regarding Responsibility for Domains of Palliative Care

Among all the providers, 80.0% felt that the medical oncologist should be the most responsible for disease management, but less so for physical concerns (44.2%) and the other care domains (2.1% to 20.0%). For these other six domains, the family physician or SPC was most often perceived as the most responsible, according to the providers.

**Figure 1:**
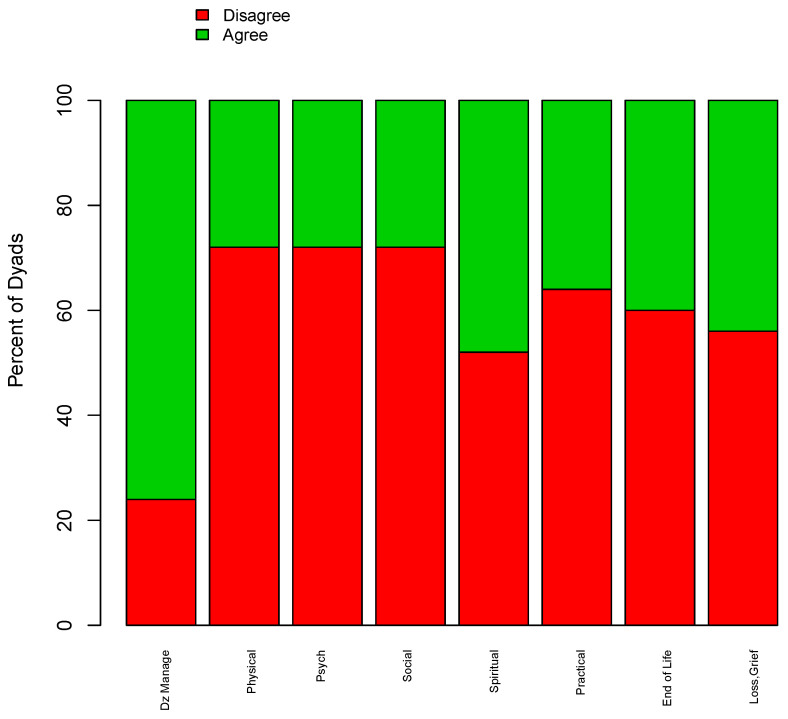
Agreement between provider/provider dyads regarding responsibility across palliative care domains regarding a mutual patient (n = 25 dyads). Figure abbreviations: Dz Manage = Disease Management, Physical = Physical Concerns, Psych = Psychological Concerns, Social = Social Concerns, Spiritual = Spiritual Concerns, Practical = Practical Concerns, End of life = Making Plans in Case Your Health Worsens, Loss, Grief = Loss and Grief.

When multiple healthcare providers answered the survey regarding the same patient, provider/provider dyads were assessed for concordance of responses. A total of 25 dyads were assessed, a maximum of one per patient (20 oncologist/family doctor, 5 oncologist/SPC provider dyads). Figure 1 shows the rate of agreement among provider dyads regarding responsibility for domains of palliative care. If both providers identified the same clinician as the most responsible, this was considered agreement. Agreement was high among providers regarding disease management (76%), with the majority identifying the medical oncologist as most responsible. Agreement was poor across all other domains (<50%).

## 4. Discussion

EIPC with usual oncology care is a new guideline-based standard [7,19] requiring the participation of non-palliative care clinicians to support all cancer patients. There is limited literature measuring collaborative care between family doctors, oncologists and SPC providers for palliative care delivery early in the trajectory of advanced cancer. In this study, we conducted surveys with patients with newly diagnosed advanced GI cancer and their providers to assess perceived importance/relevance, delivery preferences, and concordance in opinions regarding care across eight domains of EIPC. We aimed to illustrate current perspectives among patients and providers to identify opportunities to optimize EIPC and future directions for inquiry.

In the 66 patients and 95 providers represented, we found, most often, that oncologists were deemed primarily responsible for addressing needs relating to disease management and physical concerns. These were the only domains of care rated as of high importance to the patients at the time of survey completion. These domains were also rated as important by providers, but there was concordance regarding role responsibility between providers only for disease management, which was generally assigned to oncologists. For the other domains of care, most patients identified and preferred themselves as responsible for addressing related needs, which led to discordance with providers regarding perceived role responsibilities. While providers were mixed in opinion as to who should be the most responsible provider for these other domains of care, they tended to indicate that it should be the family physician or SPC. Of note, less than half of patients (41%) identified their family physician as being currently involved in their care.

The prioritization of disease management under the direction of oncologists and the importance of managing physical symptoms is expected early in the trajectory of advanced cancer. The lesser role of health care providers in other domains of care, as reported by patients, is unexpected. Most patients identified self or family as most responsible for various domains of support, even though this was not an itemized response option. This is the main reason for low concordance between patients and their providers in ascribed responsibility for domains other than disease management and physical concerns. Discordant attitudes between patients and providers regarding the relative importance of various domains of palliative care may suggest that clinicians are not always aware of the patient’s supportive care needs early in the disease trajectory [20,21,22]. It is possible that patients do not wish to rely on clinicians for some aspects of care, in which case patient activation and self-management should be incorporated into a patient-centred model of early palliative care delivery. It is also possible that patients do not believe that support is available, so they take it upon themselves. Further study is needed to understand the sources of this discordance.

Patient responses around advance care planning warrant further consideration. Patients were asked about “Making Plans in Case Your Health Worsens”, and three-quarters rated this domain as either important (24.2%), very important (27.3%), or one of the most important parts of care (21.2%), yet the vast majority ascribed responsibility to self or another non-medical provider. Poor end-of-life communication between patients and physicians impedes goal-concordant care [23] and shared decision making. Variability in patients’ illness understanding may be an obstacle to engaging with the healthcare team in this domain of EIPC [24,25,26]. Patients with advanced cancer who are unclear about their prognosis may continue to focus solely on the biomedical aspects of care rather than the other domains and, in general, death preparedness. Although all patients in our study had discussed their diagnosis and prognosis with their oncologist prior to completing the survey, we did not assess the extent to which they comprehended the seriousness of their illness. Patients with a serious illness may not seek help from their providers regarding palliative care because they lack a complete picture of their probable illness trajectory and how their health will decline over time. Even end-stage cancer patients can still be focused on the possibility of treatment and remission of their disease, particularly within different cultural backgrounds [27,28,29]. Early, high-quality goals of care discussions can be promoted via interventions like the Serious Illness Care Program [30]. This structured approach to communication emphasizes patient values and priorities in the face of serious illness and leads to improved documentation shared among care providers [31]. Such interventions could be leveraged to engage patients and families in advance care planning and goals of care discussion with healthcare providers.

Another unexpected finding in our study was the limited perceived role of the family doctor (FD). Although nearly all patients had an FD, the fact that most were not deemed to be active participants in the patient’s palliative care was surprising and required further exploration. Prior research found that patients may become disconnected from primary care upon entering the cancer system [32,33,34,35]; the patients in our study may have not yet re-engaged with their family physician. FDs have a key role in the initiation of EIPC, given the long-standing and integral care relationship they often have with their patients [36,37]. Moreover, collaborative care between FDs and oncology specialists has the potential to improve patient experience [38]. Family physicians want to be involved in palliative care but face challenges supporting patients with advanced illnesses [32,39,40]. Known barriers to collaborative care include lack of communication, role clarity, and uncertainty in knowledge [41,42,43]. In a Canadian survey, psychosocial support for patients with cancer and family members was defined as an important role for family physicians; yet, family physicians felt unequipped to engage in care along the cancer continuum due to poor communication from specialists and a lack of clearly defined roles for various care providers [33,44]. In Canada, family doctors are more likely to engage in supportive cancer care closer to end of life [45], although a survey of patients experiencing lung cancer showed a desire for more and earlier involvement of the family doctor in all facets of care [46]. Similarly, patients in Israel reported a desire for family doctor involvement while undergoing cancer treatment, but a perceived lack of communication between oncologist and family doctor led most patients to seek support mainly from specialists [47].

Discordant views regarding role responsibilities among care providers in our study highlight a need for more clearly defined expectations of each provider. A care coordination model has the potential to optimize interprofessional collaboration and patient outcomes for palliative care [48]. For example, a case conference with family doctors and specialized palliative care team members facilitates effective information exchange [49]. In a randomized trial, this strategy led to a reduction in hospitalizations and sustained performance status in cancer patients with advanced disease [50]. Such a model could be explored earlier in the cancer care trajectory and involving oncologists.

Our study has several strengths. This study is unique in considering and comparing the perspectives on the domains of EIPC both from patients and their involved providers. We based the assessment of concordance of attitudes on the individual patient and his or her direct care providers. Rather than a general survey of patients and clinicians, this pragmatic approach allows a real-world understanding of whether patients have the desired access to care and whether care is collaborative. Moreover, a global survey of clinicians about roles in the delivery of palliative care may be biased towards views on end-of-life care or patients with the highest supportive care needs. Patients with advanced cancer represent a heterogeneous population with palliative care needs ranging from minimal support to complex care. In our study, survey responses were directly related to current patient care, and many patients were early in the trajectory of serious illness. The patients included represent a spectrum of GI cancer diagnoses, prognoses, and symptom severity, contributing to generalizability.

There are some limitations to our study. We had multiple points of contact with primary oncologists to encourage patient recruitment, but a substantial proportion of potentially eligible patients were never approached or declined to participate. Our results may be vulnerable to volunteer bias. Also, the family physicians not being identified by most patients as part of their care team, and even fewer of these providers participating in this study, limited the number of dyads that could be analyzed. Although we timed the patient survey interviews to occur after their prognosis had been fully disclosed to them and discussed by their oncologist, we did not assess patients’ understanding of their illness, which could greatly influence their perspective regarding the care domains. Evaluating patients’ understanding of their life expectancy presents ethical challenges from a research perspective and requires exploration. Furthermore, our study captures patient and provider attitudes only at one time point relatively soon after the first oncology assessment for incurable cancer. It is known that the relative importance of different aspects of palliative care evolves over time [51]. Repeated surveys could offer a deeper understanding of patient and provider perspectives along the illness trajectory; however, this was not feasible within our study. We did not reach our target sample size, and findings may be seen as hypothesis-generating. This work allowed the exploration of methodologic issues with research in this area. Our cohort represents a cross-section of patients of varied ethnicities and cultures, which may have influenced their responses; however, our sample size was insufficient to analyze these potential differences. This was a single-centre study, which limits generalizability; however, our centre serves a wide catchment area, including urban and rural regions served by differing models of palliative care. In Ontario, regional disparities in the palliative care workforce are well documented, with 96% of specialized palliative care providers practicing in urban areas [52]. This indicates a larger role for family doctors in the provision of palliative care in rural areas.

## 5. Conclusions

Our results provide a real-world representation of early palliative care delivery across our region. This exploration of current attitudes among patients with advanced cancer and their healthcare providers offers a foundation for enhancing collaborative care involving an early palliative approach. If all care providers are to participate in EIPC for all cancer patients, more work is needed to address the disparate views between patients, oncology and palliative care specialists and family doctors regarding optimal care coordination. Further exploration of the patient perspectives, including across cultures, is needed. Clarifying patients’ understanding of their prognosis and illness trajectory is important to determine how this might mediate desired support from healthcare providers. A longitudinal study is also warranted on how patient preferences and attitudes change as their disease progresses.

## Figures and Tables

**Table 1:** Patient and clinician demographics and response rate (where relevant).

Patient Characteristics, n = 66		No. (%)
Age (years)	Median (min, max)	68.5 (43, 87)
Gender	n (%) Female	23 (34.9)
Diagnosis	Oesophagus	15 (22.7)
	Colon	12 (18.2)
	Pancreas	10 (15.2)
	Stomach	9 (13.6)
	Rectum	6 (9.1)
	Liver	5 (7.6)
	Other *	9 (13.6)
Employment Status	Currently Working	3 (4.6)
	Currently on Sick Leave	13 (19.7)
	Retired	48 (72.7)
	None of the Above	2 (3.0)
Relationship Status	Married	43 (65.2)
	Not Married, but in a Relationship	3 (4.6)
	Single	20 (30.3)
Children	n (%) Yes	56 (84.9)
Drug Coverage	No, I have no coverage	1 (1.5)
	Yes, I have coverage from government	34 (51.5)
	Yes, I have private insurance	31 (47.0)
Members of Healthcare Team †	Medical Oncologist	59 (89.4)
	Radiation Oncologist	17 (25.8)
	Family Doctor	27 (40.9)
	Specialized Palliative Care Provider	8 (12.1)
	Homecare Nurse	23 (34.9)
	Other	25 (37.9)
Do you have a Family Doctor	n (%) Yes	64 (97.0)
Time from prescreening to survey completion (days)	Median (range) Days	35 (0–193)
Provider Characteristics †, n = 95		
Provider Type	Family Physician	21 (22.1)
	Medical Oncologist	65 (68.4)
	Specialist Palliative Care Provider	6 (6.3)
	Radiation Oncologist	3 (3.2)
Sex	n (%) Female	44 (46.3)
Years Practicing	<1	4 (4.2)
	1–5	31 (32.6)
	6–10	15 (15.8)
	11–20	29 (30.5)
	>20	16 (16.8)
Specific Training in Palliative Care, n (%) Yes	Family Physician	6/20 (30.0)
	Medical Oncologist	39/65 (60.0)
	Specialist Palliative Care Provider	6/6 (100)
	Radiation Oncologist	1/3 (33.3)

* includes gallbladder (3), neoplasm (2), duodenum (2), bile duct (2). † members of the patient’s healthcare team that completed a provider survey; patients could have multiple providers included.

**Table 2:** Concordance of responses between patients and providers as to perceived importance (relevance) of domains of palliative care to the patient’s care.

Domain	Agreement between Patients and Physicians Who Deem Domain Most Important or Very Relevant †	Agreement between Patients and Physicians Who Deem Domain as Somewhat or Less Relevant ‡	Total Agreement
Disease Management	64/75 (85.3)	3/18 (16.7)	67/93 (72.0)
Physical Concerns	53/60 (88.3)	8/29 (27.6)	61/89 (68.5)
Psychological Concerns	16/25 (64.0)	15/66 (22.7)	31/91 (34.1)
Social Concerns	18/33 (54.6)	25/55 (45.5)	43/88 (48.9)
Spiritual Concerns	6/27 (22.2)	47/64 (73.4)	53/91 (58.2)
Practical Concerns	17/34 (50.0)	33/57 (57.9)	50/91 (54.9)
Making Plans in Case Your Health Worsens	25/47 (53.2)	25/46 (54.4)	50/93 (53.8)
Loss and Grief	13/25 (52.0)	50/65 (76.9)	63/90 (70.0)

† Numerator is the number of dyads where both patient and physician deem the domain as ‘most important or very relevant’, denominator is the number of physicians who deem the domain as ‘most important or very relevant’. ‡ Numerator is the number of dyads where both patient and physician deem the domain as ‘important, a little bit important or not at all important’, denominator is the number of physicians who deem the domain as ‘important, a little bit important or not at all important’.

**Table 3:** Patients’ preferred and perceived most responsible provider by domains of palliative care.

	Provider Type *					
Domain	Medical Oncologist	Radiation Oncologist	Family Doctor	Specialist Palliative Care Providers	Other	Total Agreement
Disease Management	38/45 (84.4)	1/1 (100)	1/3 (33.3)	1/2 (50.0)	8/15 (53.3)	49/66 (74.2)
Physical Concerns	19/26 (73.1)	1/2 (50.0)	2/3 (66.6)	7/9 (77.7)	24/26 (92.3)	53/66 (80.3)
Psychological Concerns	0/5 (0)	0/0	1/3 (33.3)	0/0	55/58 (94.8)	56/66 (84.8)
Social Concerns	0/0	0/0	3/3 (100)	1/1 (100)	62/62 (100)	66/66 (100)
Spiritual Concerns	1/1 (100)	0/0	0/0	1/1 (100)	64/64 (100)	66/66 (100)
Practical Concerns	0/0	0/0	0/0	0/0	65/66 (98.5)	65/66 (98.5)
Making Plans in Case Your Health Worsens	3/3 (100)	0/0	3/4 (75.0)	2/2 (100)	57/57 (100)	65/66 (98.5)
Loss and Grief	1/1 (100)	0/0	1/3 (33.3)	1/1 (100)	61/61 (100)	64/66 (97.0)

* Provider is preferred most responsible/provider is actual most responsible (percent).

## Data Availability

De-identified data are available from the corresponding author upon reasonable request.

## Supplementary Materials

The following supporting information can be downloaded at https://www.mdpi.com/article/10.3390/curroncol31060253/s1, Figure S1: Patient exclusion and recruitment; Table S1: Patient survey responses by domain of palliative care; Table S2: Physician survey responses by the domain of palliative care as to general importance.
