# Supplementary material for: Patient and Provider Attitudes and Preferences Regarding Early Palliative Care Delivery for Patients with Advanced Gastrointestinal Cancers: A Prospective Survey
Source: Curr Oncol. 2024 Jun 13;31(6):3329–41. doi: 10.3390/curroncol31060253 (PMC11203221; doi:10.3390/curroncol31060253)
Supplement: Supplementary file 1 [file curroncol-31-00253-s001.zip › curroncol-3016912-supplementary.pdf]

**Supplemental Tables**

**Supplemental Table S1. Patient survey responses by domain of palliative care, n = 66.**

| Variable | Response options | Domain of Palliative Care: Number (% within domain) | | | | | | | |
|---|---|---|---|---|---|---|---|---|---|
| | | **Disease Management** | **Physical Concerns** | **Psychological Concerns** | **Social Concerns** | **Spiritual Concerns** | **Practical Concern** | **Making Plans in Case Your Health Worsens** | **Loss and Grief** |
| **How important is this part of your care currently?** | Not at all important to me | 1 (1.5) | 5 (7.6) | 14 (21.2) | 17 (25.8) | 24 (36.4) | 18 (27.3) | 9 (13.6) | 18 (27.3) |
| | A little bit important to me | 4 (6.1) | 6 (9.1) | 16 (24.2) | 9 (13.6) | 15 (22.7) | 4 (6.1) | 8 (12.1) | 14 (21.2) |
| | Important to me | 8 (12.1) | 10 (15.2) | 18 (27.3) | 16 (24.2) | 8 (12.1) | 19 (28.8) | 16 (24.2) | 17 (25.8) |
| | Very important, but not the most important | 25 (37.9) | 25 (37.9) | 13 (19.7) | 13 (19.7) | 9 (13.6) | 19 (28.8) | 18 (27.3) | 15 (22.7) |
| | One of the most important parts of my care | 28 (42.4) | 20 (30.3) | 5 (7.6) | 11 (16.7) | 10 (15.2) | 6 (9.1) | 14 (21.2) | 1 (1.5) |
| | Unsure | 0 | 0 | 0 | 0 | 0 | 0 | 1 (1.5) | 1 (1.5) |
| **Who is helping you most with this part of your care?** | Medical oncologist | 46 (69.7) | 21 (31.8) | 2 (3.1) | 0 | 1 (1.5) | 0 | 3 (4.6) | 1 (1.5) |
| | Radiation oncologist | 2 (3.0) | 1 (1.5) | 0 | 0 | 0 | 0 | 0 | 0 |
| | Family doctor | 2 (3.0) | 3 (4.6) | 3 (4.6) | 3 (4.6) | 0 | 1 (1.5) | 3 (4.6) | 1 (1.5) |
| | Specialized pain and symptom team | 1 (1.5) | 7 (10.6) | 0 | 1 (1.5) | 0 | 0 | 2 (3.0) | 1 (1.5) |
| | Other | 6 (9.1) | 5 (7.6) | 1 (1.5) | 7 (10.6) | 13 (19.7) | 4 (6.1) | 3 (4.6) | 2 (3.0) |
| | Mostly family (inc spouse and friends) | 4 (6.1) | 7 (10.6) | 23 (34.9) | 24 (36.4) | 14 (19.7) | 37 (56.1) | 23 (34.9) | 28 (42.4) |
| | No one or self | 5 (7.6) | 16 (24.2) | 36 (54.6) | 29 (43.9) | 39 (59.1) | 19 (28.8) | 26 (39.4) | 26 (39.4) |
| | No preference or not sure | 0 | 2 (3.0) | 0 | 0 | 0 | 0 | 1 (1.5) | 1 (1.5) |
| | Homecare | 0 | 4 (6.1) | 0 | 0 | 0 | 5 (7.6) | 5 (7.6) | 4 (6.1) |
| | Social worker | 0 | 0 | 1 (1.5) | 2 (3.0) | 0 | 0 | 0 | 2 (3.0) |
| **Who would you like to help you the most with** | Medical oncologist | 45 (68.2) | 26 (39.4) | 5 (7.6) | 0 | 1 (1.5) | 0 | 3 (4.6) | 1 (1.5) |
| | Radiation oncologist | 1 (1.5) | 2 (3.0) | 0 | 0 | 0 | 0 | 0 | 0 |
| | Family doctor | 3 (4.6) | 3 (4.6) | 3 (4.6) | 3 (4.6) | 0 | 0 | 4 (6.1) | 3 (4.6) |
| | Specialized pain and symptom team | 2 (3.0) | 9 (13.6) | 0 | 1 (1.5) | 1 (1.5) | 0 | 2 (3.0) | 1 (1.5) |
| | Other | 4 (6.1) | 4 (6.1) | 3 (4.6) | 5 (7.6) | 14 (21.2) | 4 (6.1) | 2 (3.0) | 2 (3.0) |
| | Mostly family (inc spouse and friends) | 2 (3.0) | 7 (10.6) | 22 (33.3) | 22 (33.3) | 11 (16.7) | 29 (43.9) | 19 (28.8) | 21 (31.8) |

| | | | | | | | | | |
|---|---|---|---|---|---|---|---|---|---|
| **this part of your care?** | No one or self | 1 (1.5) | 6 (9.1) | 19 (28.8) | 29 (43.9) | 38 (57.6) | 22 (33.3) | 23 (34.9) | 25 (37.9) |
| | No preference or not sure | 8 (12.1) | 6 (9.1) | 6 (9.1) | 4 (6.1) | 0 | 2 (3.0) | 7 (10.6) | 6 (9.1) |
| | Homecare | 0 | 3 (4.6) | 0 | 0 | 0 | 9 (13.6) | 6 (9.1) | 4 (6.1) |
| | Social worker | 0 | 0 | 8 (12.1) | 2 (3.0) | 1 (1.5) | 0 | 0 | 3 (4.6) |

**Supplemental Table S2. Physician survey responses by domain of palliative care as to general importance.**

| Domain of Palliative Care | Perceived importance of domain | Family Physician (n=21) Number (%) | Medical Oncologist (n=65) Number (%) | Palliative Care Specialist (n=6) Number (%) | Radiation Oncologist (n=3) Number (%) |
|---|---|---|---|---|---|
| **Disease Management** | Not at all important | 0 | 0 | 0 | 1 (33) |
| | Minimally important | 0 | 0 | 0 | 0 |
| | Somewhat important | 2 (9.5) | 15 (23.1) | 0 | 0 |
| | Very important, but not the most important | 7 (33.3) | 9 (13.9) | 0 | 1 (33) |
| | Among the most important domains | 12 (57.1) | 41 (63.1) | 6 (100) | 1 (33) |
| **Physical** | Not at all important | 0 | 0 | 0 | 0 |
| | Minimally important | 0 | 0 | 0 | 0 |
| | Somewhat important | 1 (4.8) | 3 (4.6) | 0 | 0 |
| | Very important, but not the most important | 3 (14.3) | 9 (13.9) | 1 (17) | 1 (33) |
| | Among the most important domains | 17 (81.0) | 53 (81.5) | 5 (83) | 2 (67) |
| **Psychological** | Not at all important | 0 | 0 | 0 | 0 |
| | Minimally important | 0 | 0 | 0 | 0 |
| | Somewhat important | 0 | 2 (3.1) | 1 (17) | 0 |
| | Very important, but not the most important | 5 (23.8) | 20 (30.8) | 1 (17) | 1 (33) |
| | Among the most important domains | 15 (71.4) | 43 (66.2) | 4 (67) | 2 (67) |
| | Missing | 1 (4.8) | | | |
| **Social** | Not at all important | 0 | 0 | 0 | 0 |
| | Minimally important | 0 | 0 | 0 | 0 |
| | Somewhat important | 2 (9.5) | 9 (13.9) | 0 | 0 |
| | Very important, but not the most important | 9 (42.9) | 49 (75.4) | 5 (83) | 2 (67) |
| | Among the most important domains | 10 (47.6 | 7 (10.8) | 1 (17) | 1 (33) |

| | | | | | |
|---|---|---|---|---|---|
| **Spiritual** | Not at all important | 0 | 0 | 0 | 0 |
| | Minimally important | 0 | 1 (1.5) | 0 | 0 |
| | Somewhat important | 4 (19.1) | 28 (43.1) | 1 (17) | 1 (33) |
| | Very important, but not the most | 8 (38.1) | 29 (44.6) | 4 (67) | 1 (33) |
| | important | 9 (42.9) | 7 (10.8) | 1 (17) | 1 (33) |
| | Among the most important domains | | | | |
| **Practical** | Not at all important | 0 | 0 | 0 | 0 |
| | Minimally important | 0 | 0 | 0 | 0 |
| | Somewhat important | 1 (4.8) | 12 (18.5) | 2 (33) | 1 (33) |
| | Very important, but not the most | 8 (38.1) | 41 (63.1) | 4 (67) | 0 |
| | important | 12 (57.1) | 12 (18.5) | 0 | 2 (67) |
| | Among the most important domains | | | | |
| **End of life care/death management** | Not at all important | 0 | 0 | 0 | 0 |
| | Minimally important | 1 (4.8) | 0 | 0 | 0 |
| | Somewhat important | 0 | 2 (3.1) | 1 (17) | 2 (67) |
| | Very important, but not the most | 5 (23.8) | 14 (21.5) | 0 | 0 |
| | important | 15 (71.4) | 48 (73.9) | 5 (83) | 1 (33) |
| | Among the most important domains | | 1 (1.5) | | |
| | Missing | | | | |
| **Loss, Grief** | Not at all important | 0 | 0 | 0 | 0 |
| | Minimally important | 1 (4.8) | 1 (1.5) | 1 (17) | 0 |
| | Somewhat important | 1 (4.8) | 24 (36.9) | 2 (33) | 1 (33) |
| | Very important, but not the most | 7 (38.1) | 25 (38.5) | 3 (50) | 1 (33) |
| | important | 11 (52.4) | 15 (23.1) | 0 | 1 (33) |
| | Among the most important domains | | | | |

**Supplemental Figure**

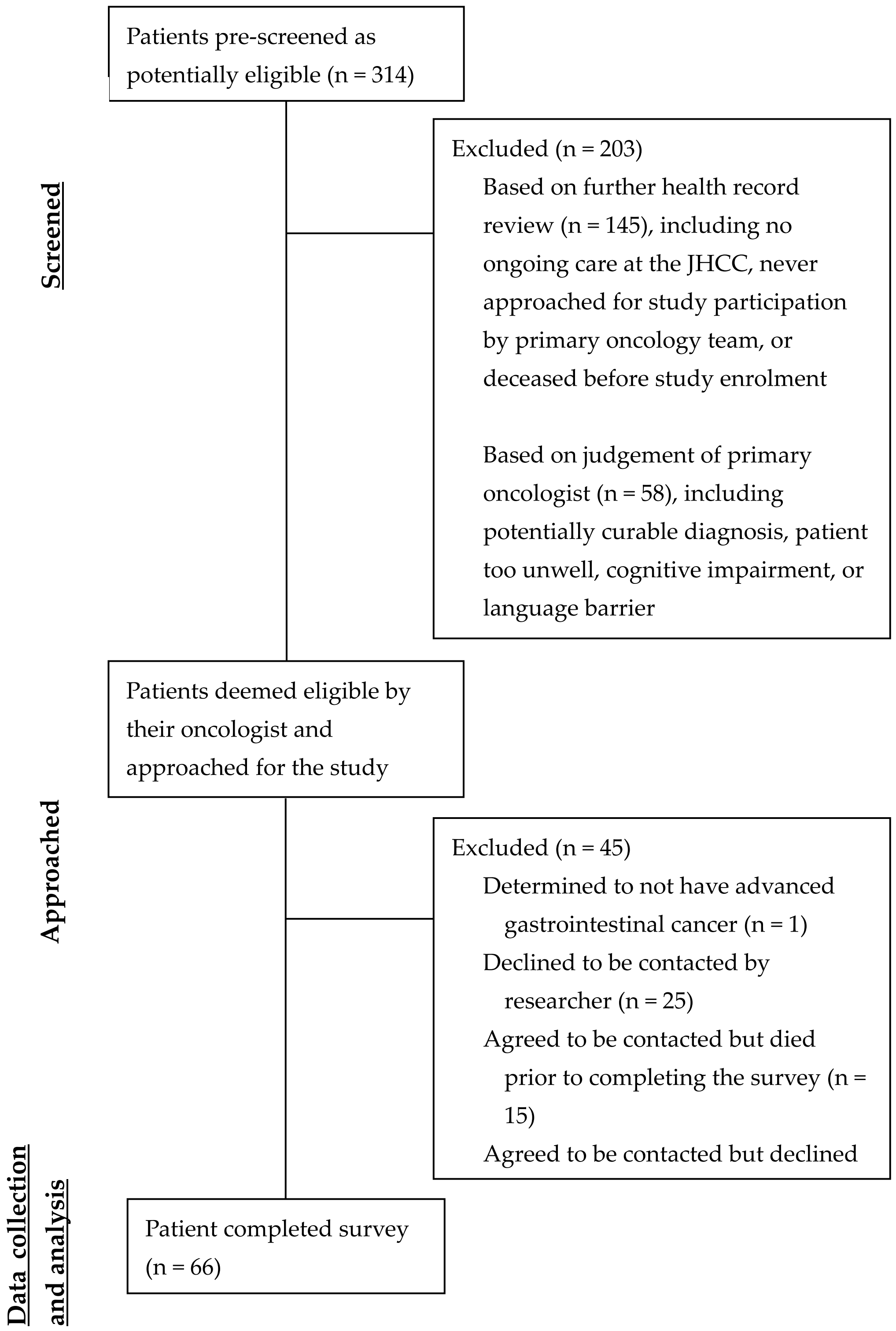


**Supplemental Figure S1.** Patient exclusion and recruitment.
